# Changes in the Epidemiology of Thoracic and Cardiovascular Diseases in Korea During the COVID-19 Pandemic: A Nationwide Analysis

**DOI:** 10.3390/jcm13237059

**Published:** 2024-11-22

**Authors:** Jung Ho Park, Hong Kyu Lee, Hyoung Soo Kim, Kunil Kim, Yong Joon Ra, Jeong Wook Kang

**Affiliations:** 1 Department of Surgery, Hallym University Sacred Heart Hospital, Anyang 14068, Republic of Korea; 2 Department of Thoracic and Cardiovascular Surgery, Hallym University Sacred Heart Hospital, Anyang 14068, Republic of Korea; 3 Department of Otorhinolaryngology-Head & Neck Surgery, Hallym University Sacred Heart Hospital, Anyang 14068, Republic of Korea

**Keywords:** coronavirus disease 2019, pandemics, thoracic diseases, pneumothorax, medical visit

## Abstract

**Background/Objectives:** There is limited evidence regarding the impact of the coronavirus disease 2019 (COVID-19) pandemic on the epidemiology of thoracic and cardiovascular diseases. This study aimed to investigate changes in medical visits for these conditions during the COVID-19 pandemic. **Methods:** We analyzed the entire Korean population (~50 million) for monthly medical visits for 15 common thoracic and cardiovascular conditions, including pneumothorax, large bullae, lung cancer, esophageal cancer, thymoma, empyema, mediastinitis, esophageal rupture, multiple rib fractures, hemothorax, rib mass, varicose vein, pectus excavatum, aortic dissection, aortic aneurysm, and valve disease from January 2019 to December 2021. Data were obtained from the Korean National Health Insurance Service using the International Classification of Disease (ICD)-10 codes. Variations in the mean monthly medical visits of 15 frequent thoracic and cardiovascular diseases before and during the COVID-19 pandemic were compared using the Mann–Whitney U test, while changes in variance were assessed using Levene’s test. **Results:** The mean monthly number of medical visits for pneumothorax and large bullae significantly decreased during the COVID-19 pandemic compared to before the pandemic (by 10.1% and 12.8%; both *p* < 0.001). On the contrary, there was a significant increase in the mean monthly counts of medical visits for lung cancer, esophageal cancer, thymoma, and valve disease diagnosis (by 6.6%, 5.3%, 8.8%, and 5.0%, respectively; all *p* < 0.05). **Conclusions:** In Korea, the number of diagnosed cases of pneumothorax significantly decreased during the COVID-19 pandemic compared to before COVID-19, while diagnoses of thoracic cancers and valve disease increased.

## 1. Introduction

The coronavirus disease 2019 (COVID-19), caused by severe acute respiratory syndrome coronavirus 2, has significantly impacted everyday life worldwide [1,2,3]. Following the World Health Organization’s declaration of COVID-19 as a pandemic, many countries implemented extensive lockdowns [4]. This pandemic has deeply affected various sectors, including health care, education, tourism, and the economy. In 2020, COVID-19 became the third major cause of death in the United States, accounting for 12.2% of all mortalities [5]. The COVID-19 pandemic impacted the entire health care system, with medical resources predominantly allocated to managing COVID-19 cases. This allocation led to significant delays in routine medical care and elective surgeries [6]. Additionally, mental health problems rose, with the prevalence of anxiety and depression increased to 44% and 62%, respectively [7]. The incidence of hemorrhagic stroke also increased during the COVID-19 pandemic [8].

In Korea, the COVID-19 pandemic influenced the incidence of various diseases. The number of patients diagnosed with cerebral infarction slightly decreased, while cases of myocardial infarction, arrhythmia, and cardiac failure significantly increased [9]. Among Korean adolescents, subjective stress levels were lower during the pandemic compared to the period before COVID-19 [10]. Regarding allergic diseases, the mean number of medical visits for allergic rhinitis, asthma, and atopic dermatitis increased during the COVID-19 pandemic compared to the pre-pandemic period [11].

However, there is a lack of studies examining the epidemiology of thoracic and cardiovascular disease during the COVID-19 pandemic. We hypothesized that trends in the occurrence of these conditions could be affected by the COVID-19 pandemic. To test this hypothesis, we compared the occurrence and variations in various thoracic and cardiovascular diseases before and during the COVID-19 pandemic. To account for seasonal variations, we analyzed the monthly incidence of these diseases.

Few studies have primarily investigated the impact of the COVID-19 pandemic on the epidemiology of thoracic and cardiovascular diseases at a national level. This study may provide valuable information regarding the effects of the COVID-19 pandemic on various thoracic and cardiovascular diseases.

## 2. Materials and Methods

### 2.1. Data Collection

The research included the entire Korean population (approximately 50 million people). This was made possible by Korea’s single obligatory national health insurance system, which covers nearly the entire population and provides medical services, including tertiary care. We collected medical records of all individuals from the Korean National Health Insurance claims database. We calculated the occurrence of thoracic and cardiovascular disease from January 1, 2019 to December 31, 2021. As the patients of COVID-19 were first recognized on January 20, 2020 in Korea and infection control measures were implemented from March 2020 onward, we defined the ‘before the COVID-19 pandemic’ period as up to February 28, 2020 and the ‘during the COVID-19 pandemic’ period as starting from March 1, 2020.

We assessed the monthly incidences of 15 common thoracic and cardiovascular diseases. Patients were identified using the following ICD-10 codes: pneumothorax (J930, J931, J938, J939), large bullae (J439), lung cancer (C349), esophageal cancer (C150, C151, C152, C153, C154, C155, C158, C159), thymoma (D150, D152, C37), empyema (J860, J869), mediastinitis (J985), esophageal rupture (K223), multiple rib fractures and hemothorax (S224, S271, S272), rib mass (D167), varicose vein (I839), pectus excavatum (Q676), aortic dissection (I710), aortic aneurysm (I719), and valve disease (I350, I352, I050, I052). The data included claim data of whole hospitals and the diagnoses of patients with unique residential registration numbers; so, it was feasible to accurately count diagnoses without duplication.

### 2.2. Statistical Analysis

Differences in the mean incidence of diseases before and during the COVID-19 pandemic were compared using the Mann–Whitney U test for non-parametric values. Differences in variance of diseases before and during the COVID-19 pandemic were compared using Levene’s test for non-parametric values. In subgroup analyses, we categorized the patients by age (<20, 20–59, and ≥60 years) and gender. Two-tailed tests were performed, and *p* values < 0.05 were regarded as statistically significant. Statistical analyses were conducted using SPSS version 22.0 (IBM, Armonk, NY, USA).

## 3. Results

Table 1 reveals a variation in the monthly counts of medical visits for thoracic and cardiovascular diseases before and during the COVID-19 pandemic. The mean monthly visits for pneumothorax and large bullae were significantly lower during the COVID-19 pandemic than before COVID-19 (10.1% and 12.8%, both *p* < 0.001). However, there was a significant increase in the diagnosis of lung cancer, esophageal cancer, thymoma, and valve disease during the COVID-19 pandemic (*p* = 0.002, 0.004, 0.016, and 0.044, respectively). Additionally, visits for mediastinitis were significantly more frequent during the COVID-19 pandemic (*p* = 0.009). The remaining nine diseases did not show a significant change in the number of visits before and during the COVID-19 pandemic (all *p* ≥ 0.05).

**Figure 1:**
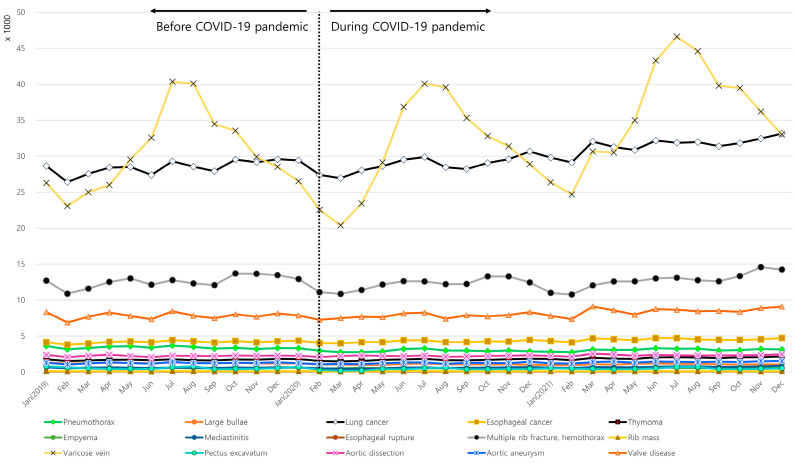
Monthly incidence of thoracic diseases during 2019, 2020, and 2021.

The number of visits for pectus excavatum and varicose vein increased during summer (July and August) both before and during the COVID-19 pandemic. On the other hand, the remaining 13 diseases showed consistent patterns throughout the year regardless of the COVID-19 pandemic (Figure 1 and Figure S1).

In subgroup analyses according to gender, trends observed in the male were similar to those of the entire population, and there was an additional significant difference in mediastinitis, aortic dissection, and aortic aneurysm. Unlike all participants, there was no significant difference in pneumothorax and valve disease in women, while there was an additional significant difference in pneumothorax (Table 2).

Regarding age groups, the <20-year-old group showed a lower incidence of medical visits for pneumothorax and large bullae (both *p* < 0.001, Table 3). Conversely, there was a statistically significant increase (*p* = 0.002) in visits for lung cancer, despite the small number of visits (Table 3). The 20 to 59-year-old group followed trends similar to the overall population, with an additional statistically significant difference in esophageal rupture (Table 3). In the ≥60-year-old group, there was a higher incidence of medical visits for lung cancer, esophageal cancer, thymoma, mediastinitis, varicose vein, aortic aneurysm, and valve disease during COVID-19 than before COVID-19 (all *p* < 0.05, Table 3).

## 4. Discussion

Kirun et al. reported effects of the COVID-19 pandemic on cardiac surgery practice and outcomes in South India. There was a dramatic decrease in cardiac surgical volume during the COVID-19 pandemic, although surgical outcomes remained unaffected [12]. Our study may be the first to analyze the impact of the COVID-19 pandemic on thoracic and cardiovascular disease using the Korean National Health Insurance claim database. The mean monthly medical visits for pneumothorax were lower during the COVID-19 pandemic than before. Meanwhile, the number of visits for lung cancer, esophageal cancer, thymoma, and valve disease increased during the COVID-19 pandemic compared to before, although the absolute increase in these visits was less than 10% (6.6%, 5.3%, 8.8%, and 5.0%, respectively), indicating limited clinical significance. Additionally, there was no significant difference in visits for empyema, mediastinitis, esophageal rupture, multiple rib fractures, rib mass, varicose vein, pectus excavatum, aortic dissection, and aortic aneurysm before COVID-19 and during the COVID-19 pandemic periods.

There are several probable explanations for the decrease in the visits for pneumothorax during the COVID-19 pandemic. First, reduced exposure to air pollutants during this period could have contributed to the decline. With the adoption of social distancing measures, air pollution levels, especially those of particulate matter, decreased significantly [13]. In a study by Han et al. [14], particulate matter and carbon monoxide were identified as potential risk factors for primary spontaneous pneumothorax. The widespread adoption of mask-wearing, driven by heightened awareness during the COVID-19 pandemic may have reduced exposure to these environmental risk factors [15]. Second, patients were hesitant to seek hospital care due to anxiety of infection, which may have affected the accessibility of the diagnostic and treatment services [16]. Health care utilization decreased overall during the pandemic, especially among patients with mild conditions [17]. In one retrospective study, cases of pneumothorax during the pandemic were found to be more severe and complicated than those seen prior to the pandemic [18]. It is possible that cases of pneumothorax with mild symptoms were overlooked during this period.

Although not clinically significant, the frequency of medical visits for lung cancer, esophageal cancer, thymoma, and valve disease during the COVID-19 pandemic increased. The medical visits for lung cancer continued to rise steadily, independent of the pandemic. This finding is somewhat unexpected, given the reduced activity of cancer screening programs during this period. Decreased lung cancer screening led to fewer diagnoses of lung cancer in the United States [19], England [20], and Canada [21]. While lung cancer screenings significantly declined during the pandemic [16], the incidence of lung cancer in Korea was not markedly affected. However, there is evidence that delayed diagnosis led to the upstaging of lung cancer [22]. Another potential explanation for the observed increase in lung cancer cases could be the more frequent use of chest imaging during the pandemic, which may have led to incidental lung cancer detection [23]. This explanation aligns with the concurrent increase in thymoma cases, a condition often identified through chest imaging [24].

Thoracic and cardiovascular diseases that did not show significant changes in frequency before and during the COVID-19 pandemic were generally severe diseases, which limited the impacts of fears related to COVID-19 on medical visits.

Previous studies have reported changes in the incidences of various diseases during the COVID-19 pandemic in Korea. Cardiovascular conditions such as myocardial infarction, heart failure, and arrhythmia increased during the COVID-19 pandemic, as did allergic diseases such as allergic rhinitis, atopic dermatitis, and asthma [9,11]. Conversely, the number of medical visits for mental illness—including depressive, panic, bipolar, and anxiety disorder—increased during the COVID-19 pandemic [25]. However, in otolaryngology, medical visits for infectious diseases like acute tonsillitis, influenza, peritonsillar, and epipharyngeal and parapharyngeal abscess decreased during the COVID-19 pandemic [26]. Furthermore, newly diagnosed cases of cancers, including gastric, colon, hepatic, breast, and uterine cervix cancer, were less frequently diagnosed during the COVID-19 period, likely due to reduced access to preventive services and delays in medical screening exams [27].

Providing basic medical services is difficult during a pandemic period. A well-designed framework, including perioperative examinations, research activities, resident training, and ethical considerations, is essential to maintain thoracic surgery services during a pandemic [28]. Many countries have implemented strategies to minimize delays in the diagnosis and treatment of thoracic and cardiovascular diseases during the COVID-19 pandemic [28,29,30].

This study is a large population study based on real-world data that compares the mean and variance of the count of medical visits of thoracic and cardiovascular diseases before and during the COVID-19 pandemic. Due to the Korean health insurance system, which uses individual resident registration numbers, the potential for data loss or overlap was minimal. The findings offer insights into the relationship between epidemic and thoracic cardiovascular diseases. However, there are several limitations. First, the study population was limited to Korea. Results can vary when applied to different health care systems. Regional differences in viral characteristics may alter the clinical manifestations of COVID-19 [31]. Second, this study relied on the national health claim codes, meaning cases where patients who did not visit a health care facility were not noticed. Third, we did not assess the number of diagnoses based on the health care setting, such as whether diagnoses were enrolled at a primary clinic or a tertiary hospital. Future research should include longitudinal follow-up with detailed treatment records to better understand the long-term effects of the COVID-19 pandemic on thoracic and cardiovascular diseases.

## 5. Conclusions

In summary, the incidence of pneumothorax significantly decreased during the COVID-19 pandemic in Korea, which may be attributable to reduced exposure to air pollutants and decreased health care utilization. The diagnoses of thoracic cancers and valve diseases increased, while most other severe thoracic and cardiovascular conditions remained unchanged.

## Figures and Tables

**Table 1:** The mean and standard deviation of monthly numbers of medical visit for thoracic diseases before and during the COVID-19 pandemic, and their difference.

Diseases	Before COVID-19		During COVID-19		*p*-Values of Difference	
	Mean	SD	Mean	SD	Mean	Variance
Pneumothorax	3367.7	196.7	3026.4	176.8	<0.001 *	0.727
Large bullae	1229.1	76.0	1072.0	62.6	<0.001 *	0.451
Lung cancer	28,410.3	966.5	30,306.8	1704.1	0.002 *	0.144
Esophageal cancer	4172.9	166.1	4395.8	220.7	0.004 *	0.249
Thymoma	1677.9	91.2	1825.7	188.2	0.016 *	0.064
Empyema	558.1	45.9	570.5	41.2	0.399	0.226
Mediastinitis	605.3	43.0	658.3	107.0	0.067	0.009 ^†^
Esophageal rupture	47.2	5.8	44.7	6.2	0.328	0.178
Multiple rib fractures, hemothorax	12,490.4	876.7	12,533.5	967.0	0.948	0.656
Rib mass	92.3	11.3	92.4	14.0	0.961	0.459
Varicose vein	29,880.2	5688.3	33,994.5	6998.6	0.080	0.761
Pectus excavatum	545.2	157.0	452.4	129.4	0.062	0.943
Aortic dissection	2239.8	102.0	2295.0	107.1	0.136	0.522
Aortic aneurysm	1243.6	100.3	1323.1	123.9	0.067	0.112
Valve disease	7790.0	446.3	8181.1	532.0	0.044 *	0.346

* Mann–Whitney U test, significance at <0.05. ^†^ Levene’s test in non-parametric data, significance at <0.05.

**Table 2:** The mean and standard deviation of monthly numbers of medical visit for thoracic diseases before and during the COVID-19 pandemic, and their difference in the subgroup by sex.

Diseases	Before COVID-19		During COVID-19		*p*-Values of Difference	
	Mean	SD	Mean	SD	Mean	Variance
Men						
Pneumothorax	2870.1	173.2	2546.1	143.3	<0.001 *	0.644
Large bullae	1079.1	71.2	936.1	54.8	<0.001 *	0.448
Lung cancer	17,728.9	578.4	18,705.8	917.3	0.003 *	0.134
Esophageal cancer	3784.9	144.6	3955.1	188.7	0.014 *	0.216
Thymoma	896.9	56.7	979.8	93.4	0.009 *	0.076
Empyema	447.6	37.8	450.9	31.5	0.697	0.233
Mediastinitis	309.4	25.1	346.7	57.4	0.035 *	0.011 ^†^
Esophageal rupture	34.6	5.6	34.2	4.4	0.948	0.164
Multiple rib fractures, hemothorax	6946.9	485.9	7071.9	486.4	0.475	0.681
Rib mass	52.3	7.9	52.2	11.7	0.845	0.461
Varicose vein	9375.9	1430.7	10,332.3	1861.5	0.112	0.771
Pectus excavatum	438.5	128.5	366.9	105.1	0.119	0.962
Aortic dissection	1334.5	69.9	1390.5	59.6	0.014 *	0.723
Aortic aneurysm	801.1	63.2	871.0	85.2	0.016 *	0.144
Valve disease	2969.9	177.7	3166.3	207.0	0.010 *	0.386
Women						
Pneumothorax	497.6	34.1	480.3	45.7	0.205	0.348
Large bullae	150.0	17.1	135.9	18.8	0.030 *	0.545
Lung cancer	10,681.4	394.5	11,601.0	792.3	0.001 *	0.156
Esophageal cancer	388.0	25.9	440.7	37.7	<0.001 *	0.337
Thymoma	781.0	47.1	845.9	98.7	0.034 *	0.051
Empyema	110.6	13.7	119.7	13.0	0.119	0.249
Mediastinitis	295.9	24.6	311.5	52.7	0.390	0.008 ^†^
Esophageal rupture	12.6	1.9	10.5	3.7	0.058	0.282
Multiple rib fractures, hemothorax	5543.5	411.3	5461.6	498.1	0.516	0.674
Rib mass	40.0	5.8	40.1	6.5	0.819	0.460
Varicose vein	20,504.3	4272.1	23,662.2	5148.3	0.074	0.759
Pectus excavatum	106.7	29.4	85.5	26.2	0.030 *	0.928
Aortic dissection	905.3	44.1	904.5	53.0	0.548	0.745
Aortic aneurysm	442.5	46.2	452.2	46.4	0.506	0.119
Valve disease	4820.1	272.4	5014.8	328.1	0.105	0.331

* Mann–Whitney U test, significance at <0.05. ^†^ Levene’s test in non-parametric data, significance at <0.05.

**Table 3:** The mean and standard deviation of monthly numbers of medical visit for thoracic diseases before and during the COVID-19 pandemic, and their difference in the subgroup by age.

Diseases	Before COVID-19		During COVID-19		*p*-Values of Difference	
	Mean	SD	Mean	SD	Mean	Variance
Age 0–19 years old						
Pneumothorax	933.6	88.5	784.0	101.6	<0.001 *	<0.001 ^†^
Large bullae	400.8	40.5	330.6	41.1	<0.001 *	<0.001 ^†^
Lung cancer	3.9	0.9	6.3	2.4	0.002 *	0.001 ^†^
Esophageal cancer	0.0	0.0	0.0	0.0	1.000	0.000 ^†^
Thymoma	18.4	6.3	16.5	5.6	0.363	0.343
Empyema	12.4	2.6	11.2	2.7	0.128	0.178
Mediastinitis	24.4	6.9	23.1	7.3	0.569	0.588
Esophageal rupture	0.8	1.2	0.9	0.9	0.540	0.828
Multiple rib fractures, hemothorax	73.6	21.8	74.7	25.1	0.948	0.888
Rib mass	10.6	4.6	8.8	3.3	0.209	0.190
Varicose vein	151.9	39.4	151.5	36.9	0.974	0.978
Pectus excavatum	443.3	145.8	357.9	114.8	0.052	0.059
Aortic dissection	1.5	1.0	1.6	1.0	0.824	0.686
Aortic aneurysm	4.1	2.0	2.7	1.2	0.068	0.013 ^†^
Valve disease	1.1	4.0	0.0	0.0	0.210	0.215
Age 20–59 years old						
Pneumothorax	1530.9	71.0	1364.6	69.4	<0.001 *	<0.000 ^†^
Large bullae	562.5	31.1	488.0	30.7	<0.001 *	<0.000 ^†^
Lung cancer	6088.0	172.0	5953.6	195.5	0.034 *	0.043 ^†^
Esophageal cancer	779.9	31.1	732.0	27.7	<0.001 *	<0.000 ^†^
Thymoma	895.2	50.6	934.6	93.3	0.131	0.157
Empyema	190.1	18.5	179.7	17.7	0.168	0.103
Mediastinitis	325.9	32.6	359.2	66.5	0.091	0.091
Esophageal rupture	21.7	3.7	19.0	3.1	0.022 *	0.021 ^†^
Multiple rib fractures, hemothorax	5766.1	360.6	5510.5	366.1	0.067	0.048 ^†^
Rib mass	58.2	8.9	56.7	10.0	0.581	0.653
Varicose vein	18,765.0	3824.8	20,334.0	4175.0	0.230	0.264
Pectus excavatum	101.4	13.5	93.8	19.5	0.211	0.210
Aortic dissection	829.9	41.8	827.0	31.4	0.685	0.819
Aortic aneurysm	210.4	23.7	214.6	23.7	0.604	0.604
Valve disease	1659.1	121.1	1472.9	65.9	<0.001 *	<0.000 ^†^
Age ≥ 60 years old						
Pneumothorax	903.2	60.4	877.9	48.1	0.189	0.172
Large bullae	265.8	18.7	253.4	19.2	0.053	0.066
Lung cancer	22,318.4	833.3	24,346.9	1534.6	<0.001 *	<0.000 ^†^
Esophageal cancer	3393.0	140.7	3663.9	203.7	0.001 *	<0.000 ^†^
Thymoma	764.3	44.7	874.6	97.7	0.001 *	<0.000 ^†^
Empyema	355.6	34.1	379.6	33.8	0.062	0.046 ^†^
Mediastinitis	254.9	12.9	276.0	40.7	0.027 *	0.071
Esophageal rupture	24.7	4.5	24.9	4.4	0.883	0.922
Multiple rib fractures, hemothorax	6650.6	557.6	6948.3	621.9	0.119	0.155
Rib mass	23.5	5.2	26.8	4.9	0.107	0.060
Varicose vein	10,963.4	1862.4	13,509.0	2999.9	0.006 *	0.008 ^†^
Pectus excavatum	0.5	0.9	0.6	1.0	0.530	0.677
Aortic dissection	1408.4	64.6	1466.4	80.8	0.052	0.030 ^†^
Aortic aneurysm	1029.1	82.7	1105.8	103.9	0.043 *	0.026 ^†^
Valve disease	6120.9	346.6	6699.0	512.9	0.002 *	0.001 ^†^

* Mann–Whitney U test, significance at <0.05. ^†^ Levene’s test in non-parametric data, significance at <0.05.

## Data Availability

Data are available from the authors upon request.

## Supplementary Materials

The following supporting information can be downloaded at: https://www.mdpi.com/article/10.3390/jcm13237059/s1, Figure S1: Comparison of thoracic disease incidence rates in 2019, 2020, and 2021.
