# Supplementary material for: Changes in the Epidemiology of Thoracic and Cardiovascular Diseases in Korea During the COVID-19 Pandemic: A Nationwide Analysis
Source: J Clin Med. 2024 Nov 22;13(23):7059. doi: 10.3390/jcm13237059 (PMC11641859; doi:10.3390/jcm13237059)
Supplement: Supplementary file 1 [file jcm-13-07059-s001.zip › jcm-3301732-supplementary.pdf]

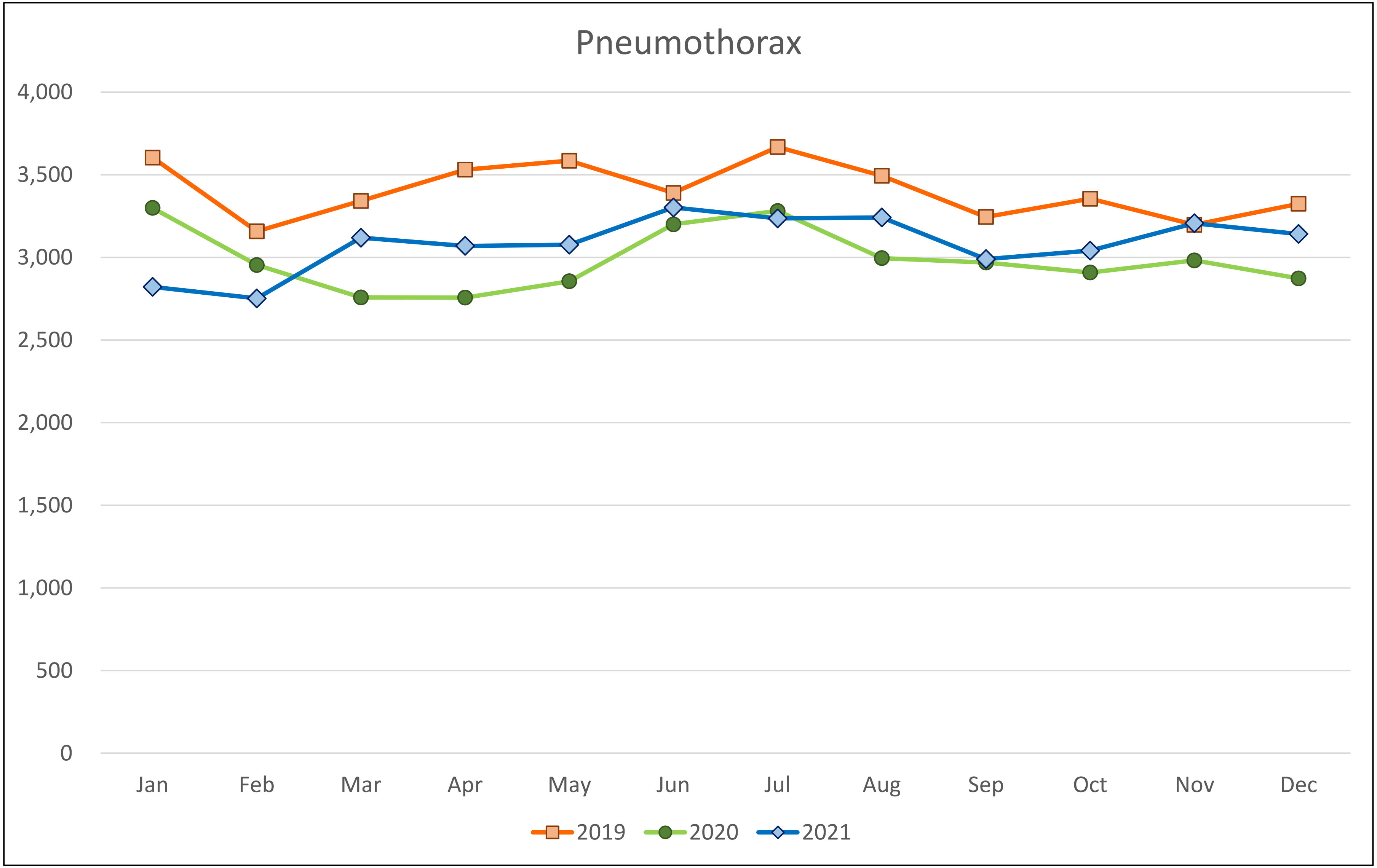

Pneumothorax
4,000
3,500
3,000
2,500
2,000
1,500
1,000
500
0
Jan
Feb
Mar
Apr
May
Jun
Jul
Aug
Sep
Oct
Nov
Dec
2019
2020
2021

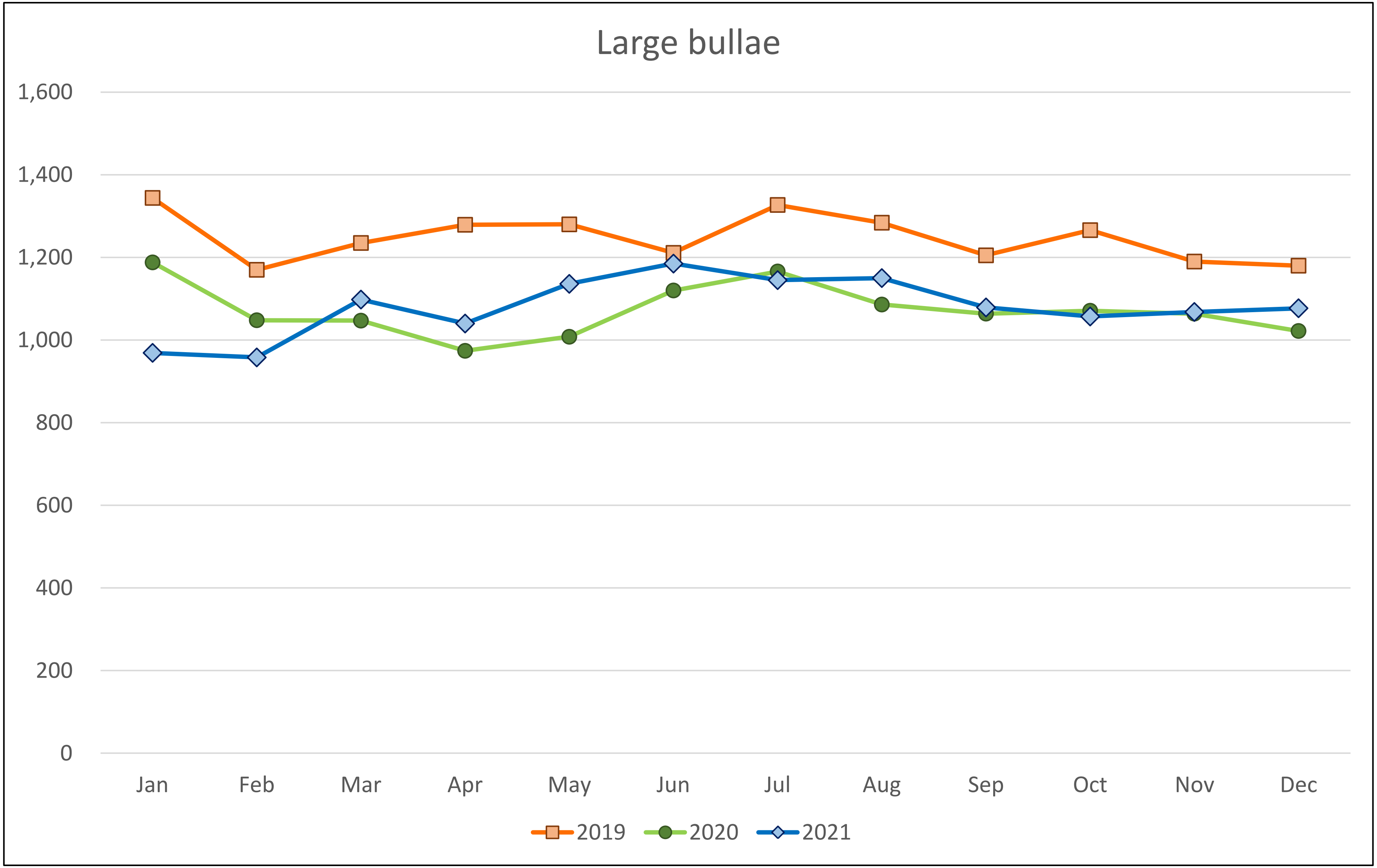

Large bullae
1,600
1,400
1,200
1,000
800
600
400
200
0
Jan
Feb
Mar
Apr
May
Jun
Jul
Aug
Sep
Oct
Nov
Dec
2019
2020
2021

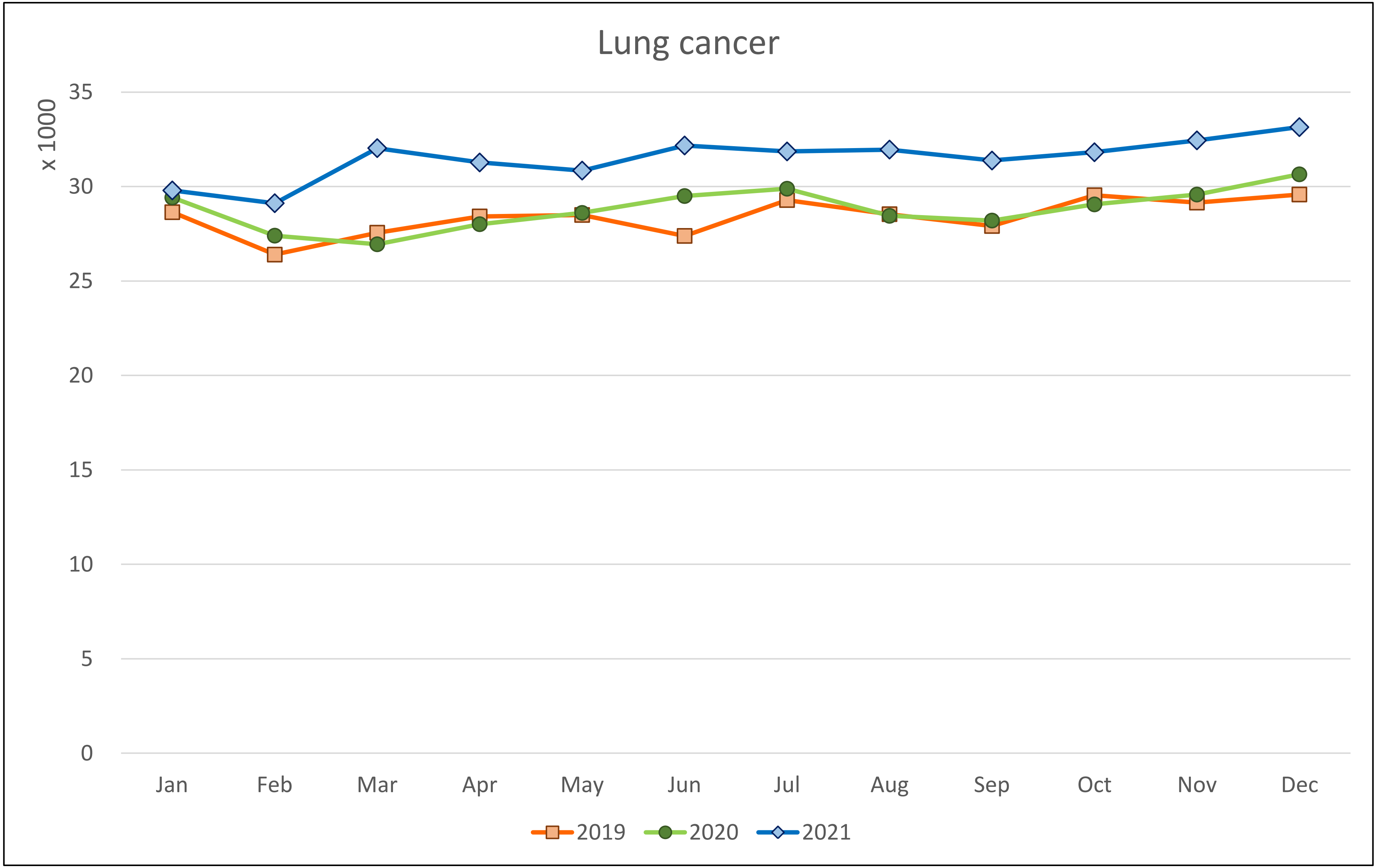

Lung cancer
x 1000
35
30
25
20
15
10
5
0
Jan
Feb
Mar
Apr
May
Jun
Jul
Aug
Sep
Oct
Nov
Dec
2019
2020
2021

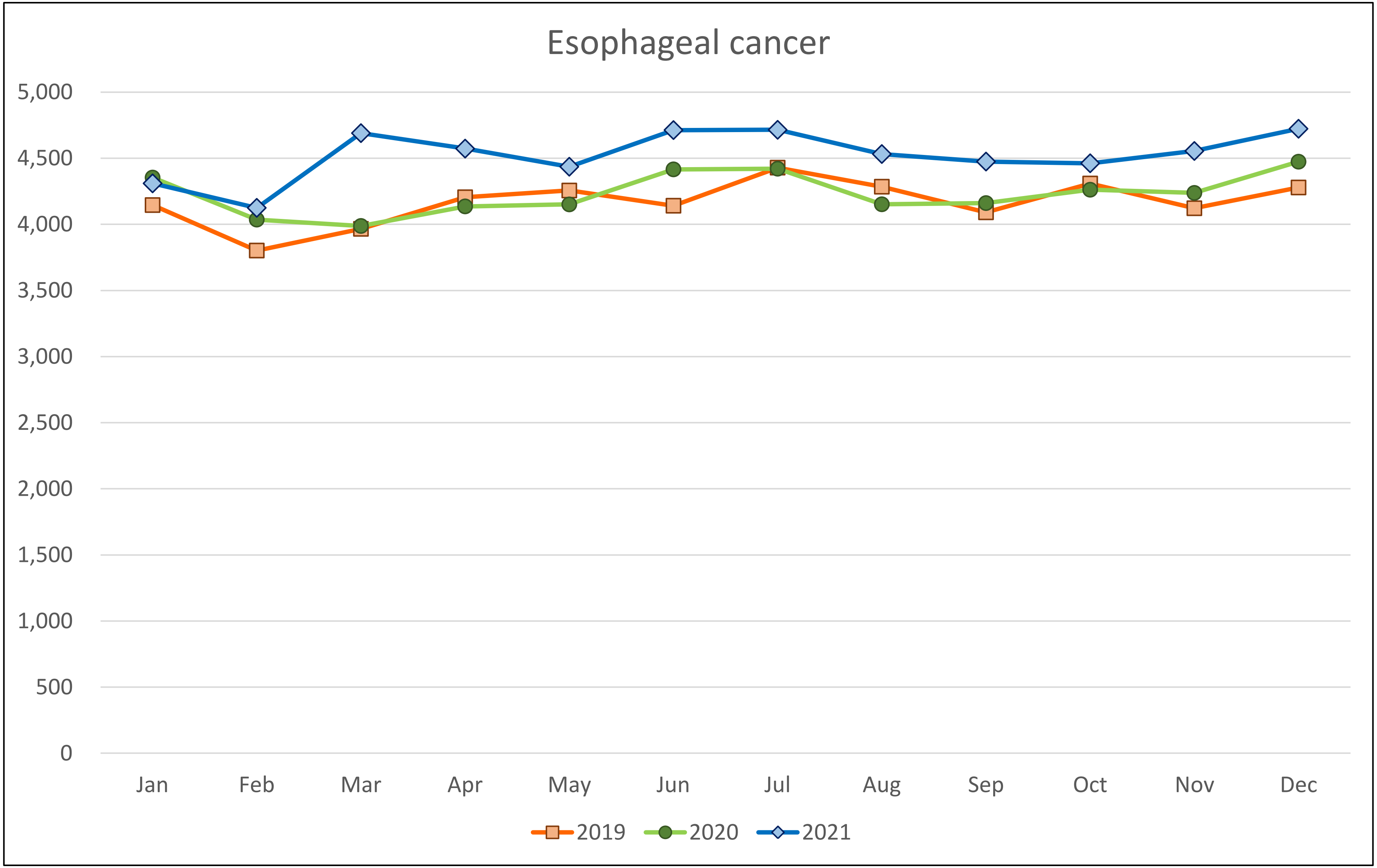

Esophageal cancer
5,000
4,500
4,000
3,500
3,000
2,500
2,000
1,500
1,000
500
0
Jan
Feb
Mar
Apr
May
Jun
Jul
Aug
Sep
Oct
Nov
Dec
2019
2020
2021

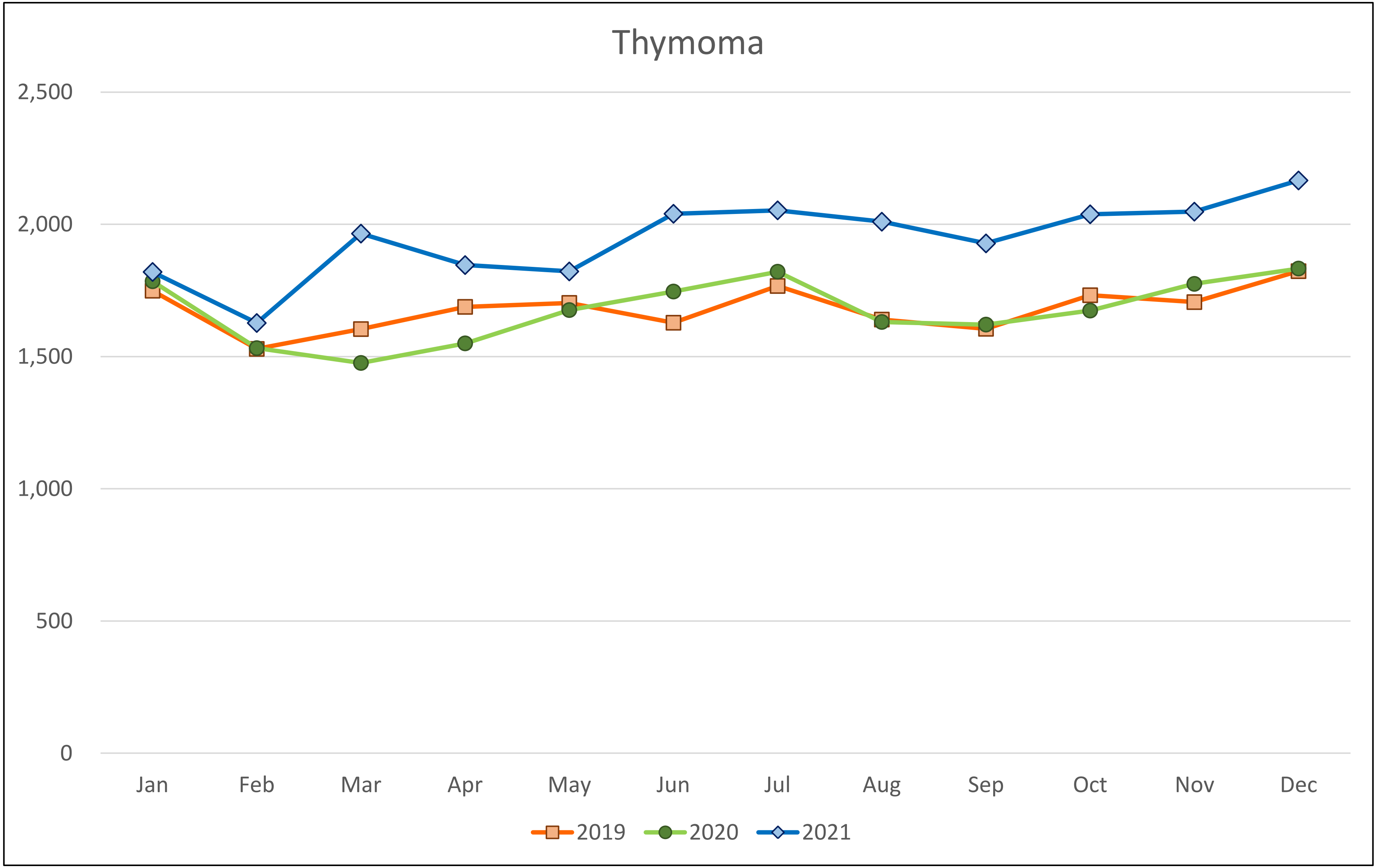

Thymoma
2,500
2,000
1,500
1,000
500
0
Jan
Feb
Mar
Apr
May
Jun
Jul
Aug
Sep
Oct
Nov
Dec
2019
2020
2021

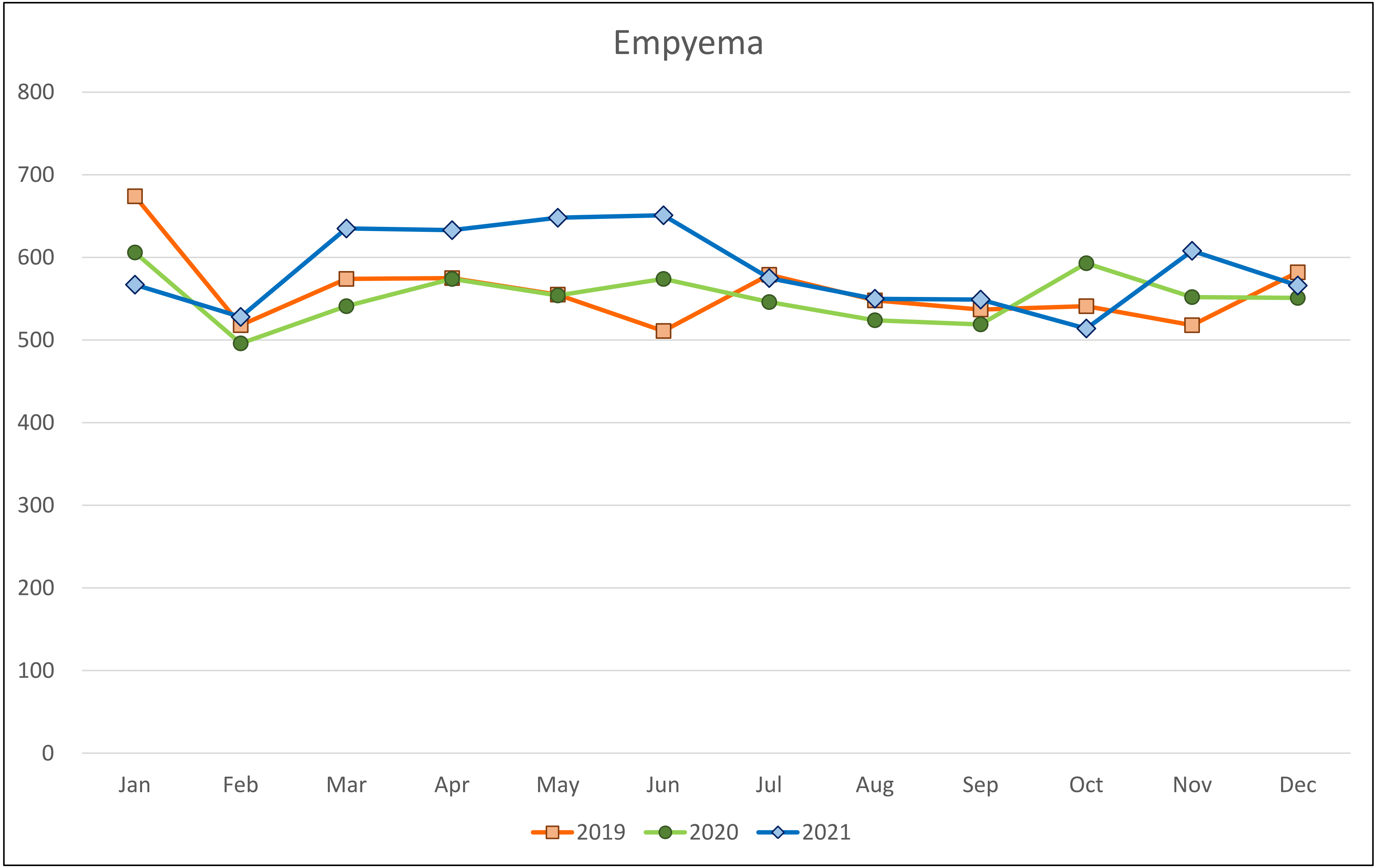
Empyema
800
700
600
500
400
300
200
100
0
Jan
Feb
Mar
Apr
May
Jun
Jul
Aug
Sep
Oct
Nov
Dec
2019
2020
2021

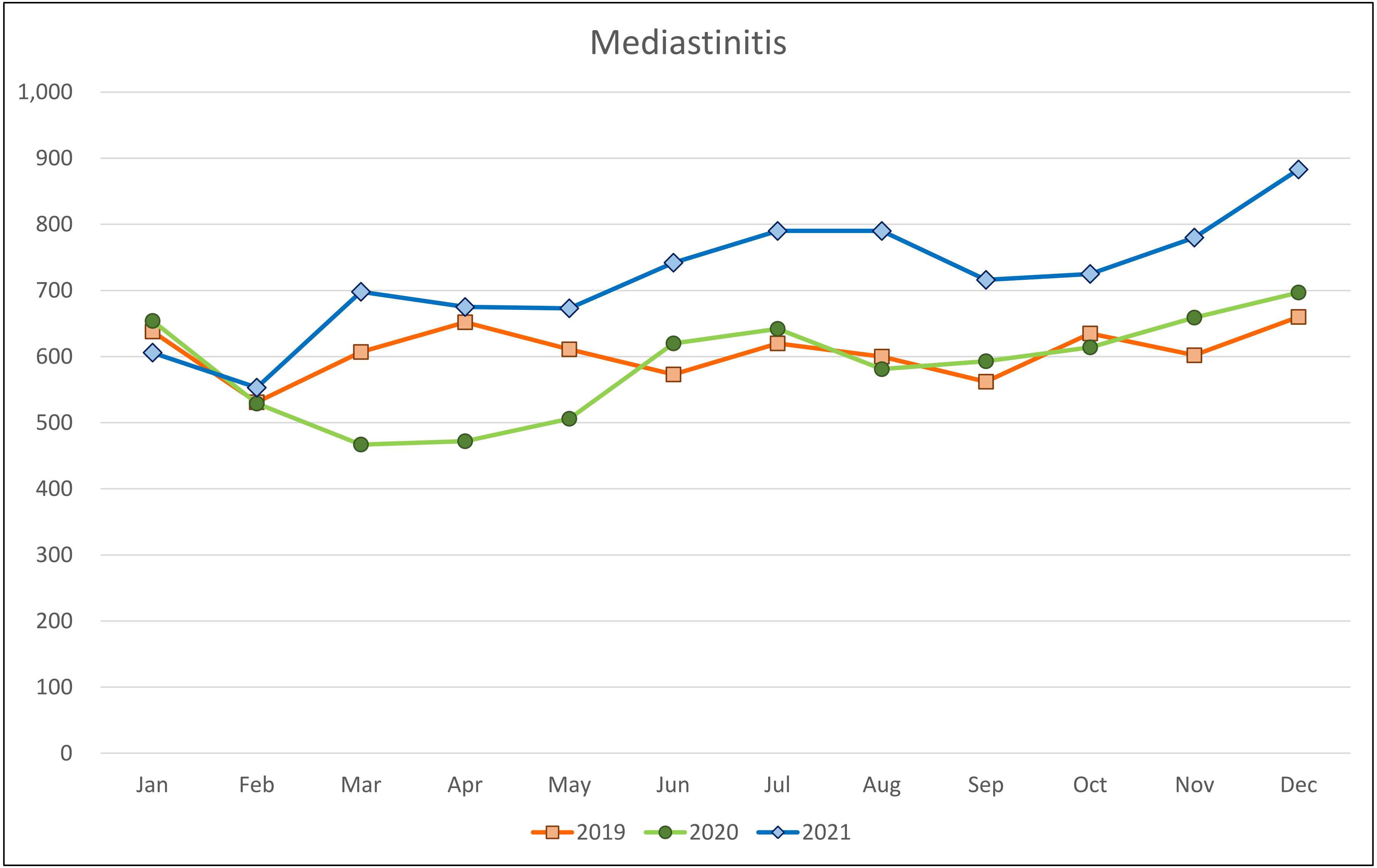

Mediastinitis
1,000
900
800
700
600
500
400
300
200
100
0
Jan
Feb
Mar
Apr
May
Jun
Jul
Aug
Sep
Oct
Nov
Dec
2019
2020
2021

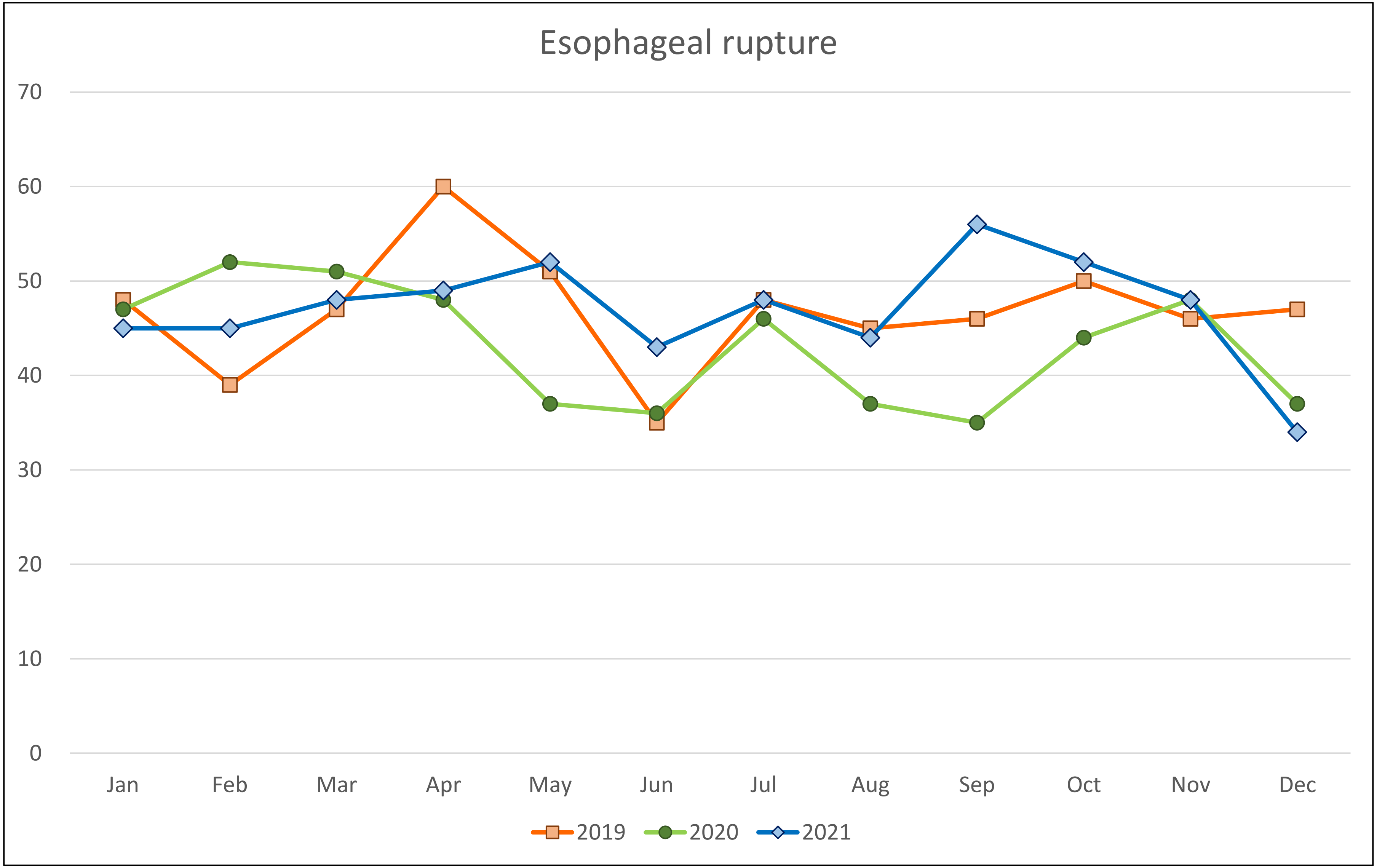
Esophageal rupture
70
60
50
40
30
20
10
0
Jan
Feb
Mar
Apr
May
Jun
Jul
Aug
Sep
Oct
Nov
Dec
2019
2020
2021

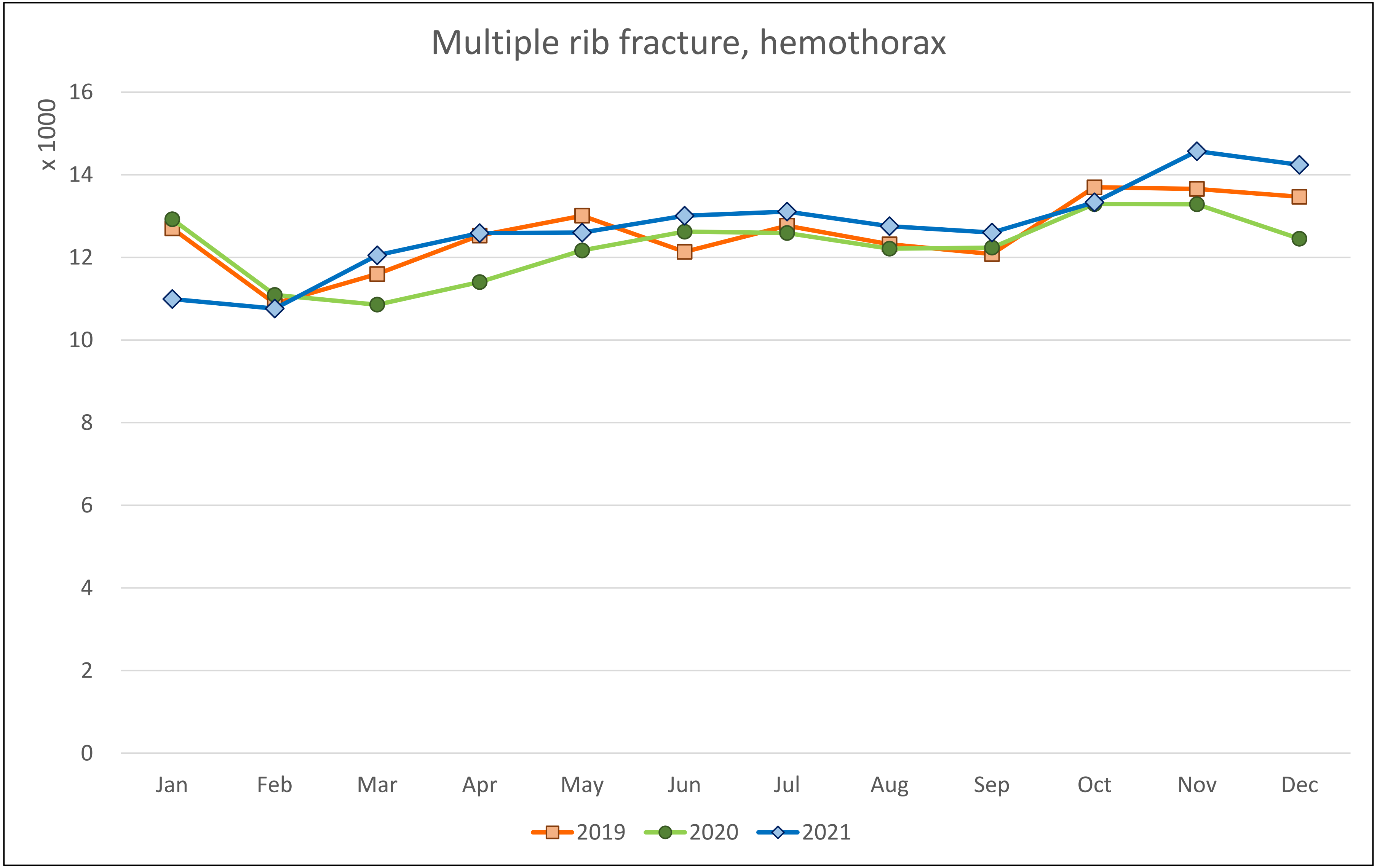

Multiple rib fracture, hemothorax
x 1000
16
14
12
10
8
6
4
2
0
Jan
Feb
Mar
Apr
May
Jun
Jul
Aug
Sep
Oct
Nov
Dec
2019
2020
2021

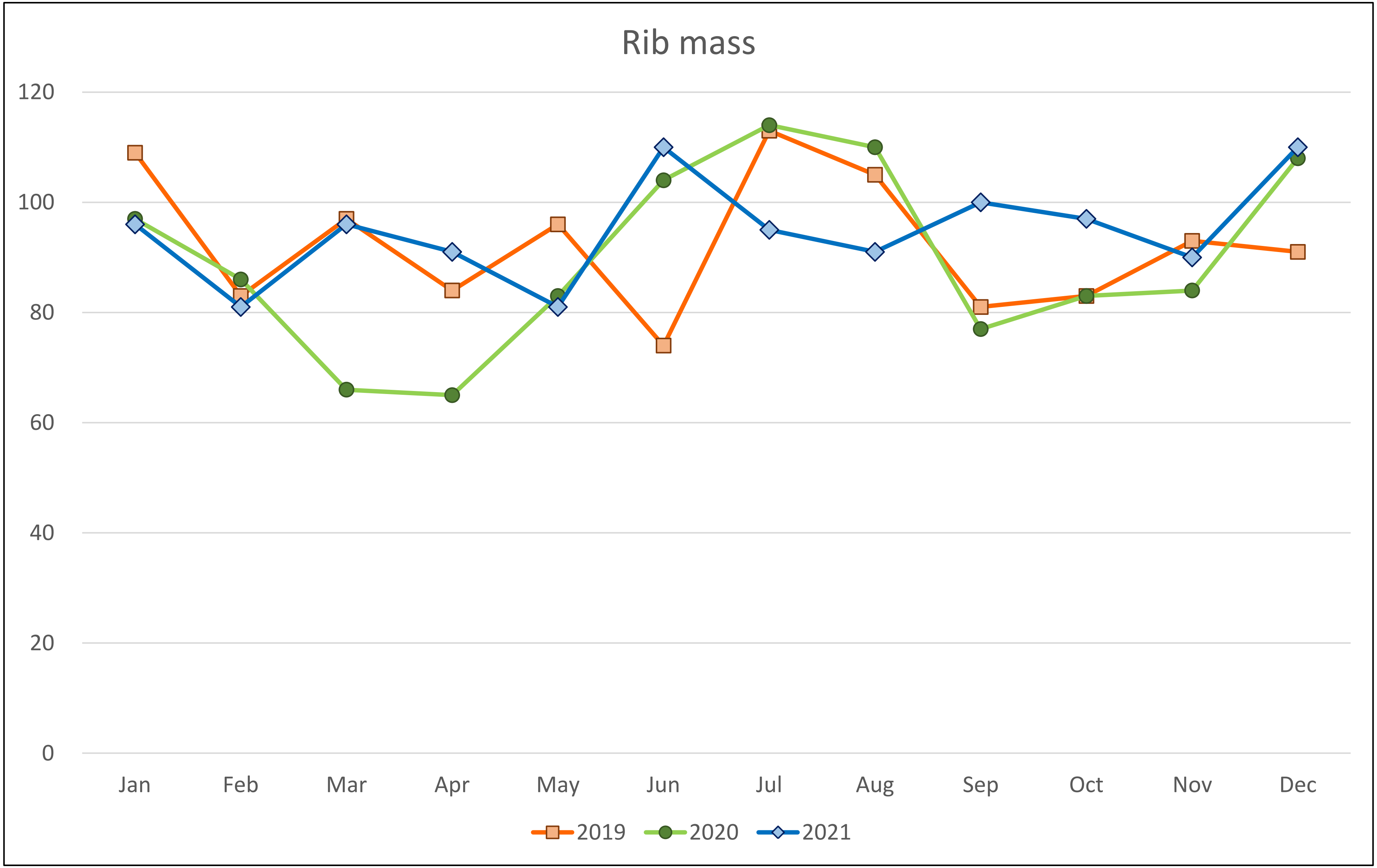
Rib mass
120
100
80
60
40
20
0
Jan
Feb
Mar
Apr
May
Jun
Jul
Aug
Sep
Oct
Nov
Dec
2019
2020
2021

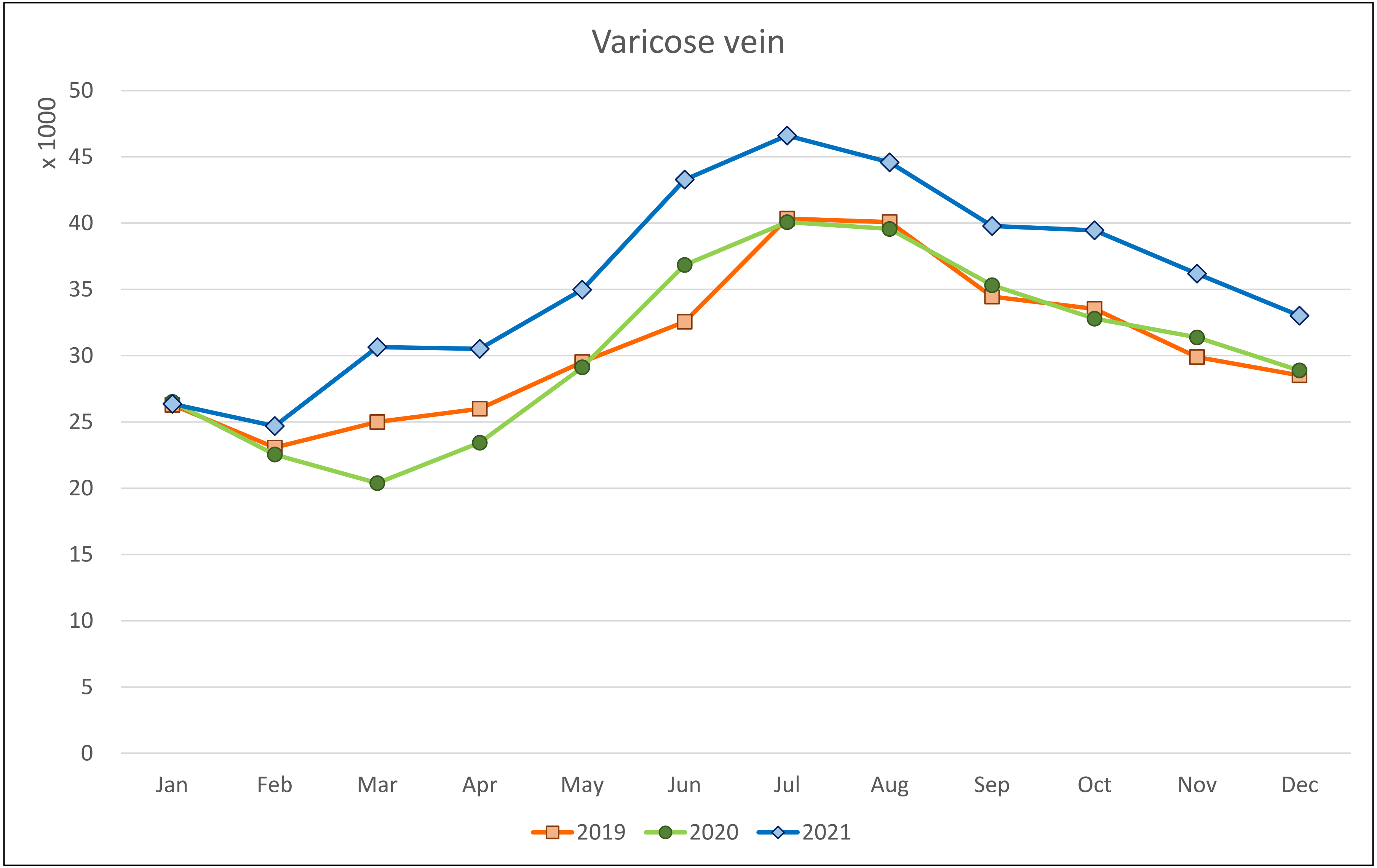

Varicose vein
x 1000
0
5
10
15
20
25
30
35
40
45
50
Jan
Feb
Mar
Apr
May
Jun
Jul
Aug
Sep
Oct
Nov
Dec
2019
2020
2021

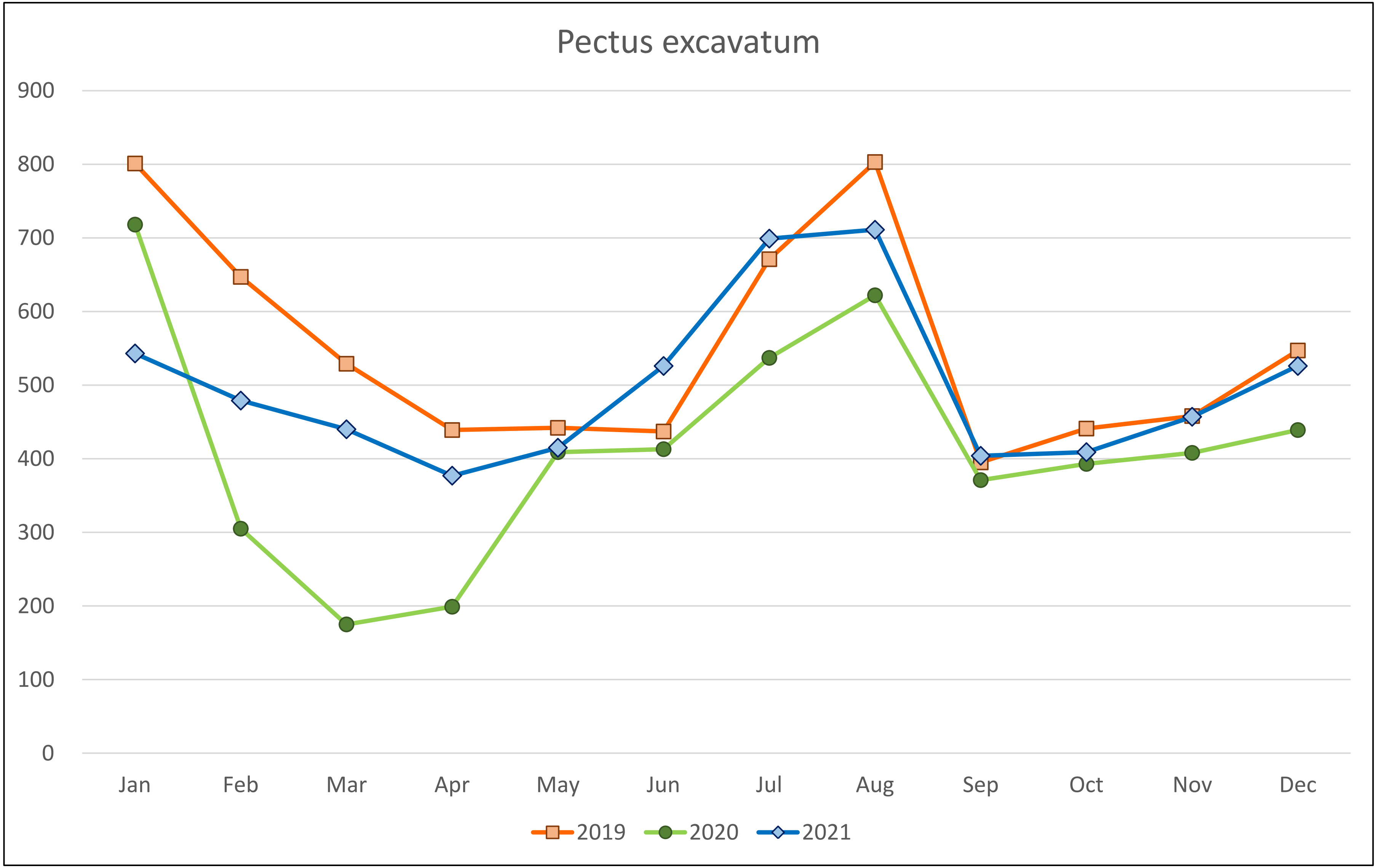
Pectus excavatum
900
800
700
600
500
400
300
200
100
0
Jan
Feb
Mar
Apr
May
Jun
Jul
Aug
Sep
Oct
Nov
Dec
2019
2020
2021

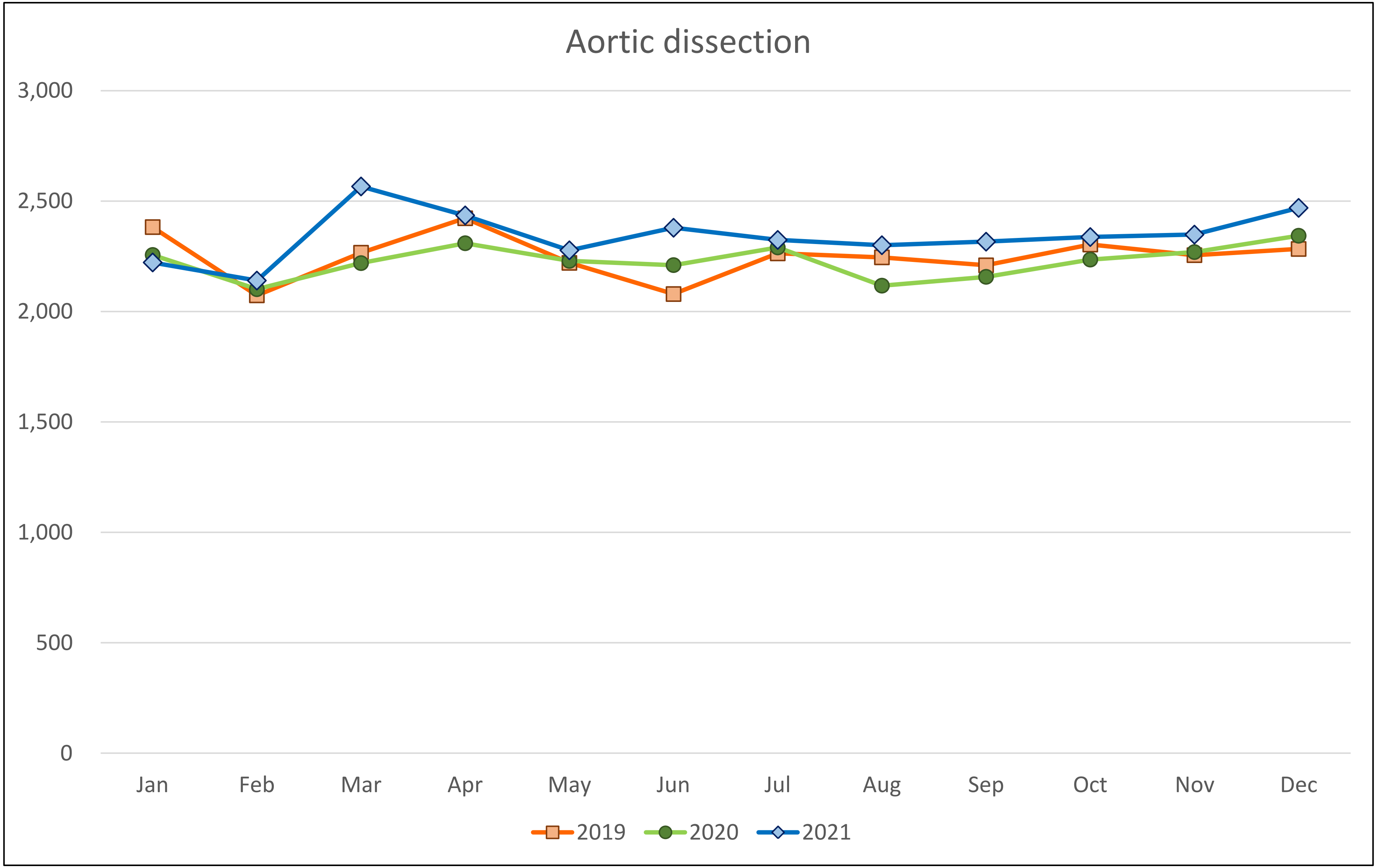

Aortic dissection
3,000
2,500
2,000
1,500
1,000
500
0
Jan
Feb
Mar
Apr
May
Jun
Jul
Aug
Sep
Oct
Nov
Dec
2019
2020
2021

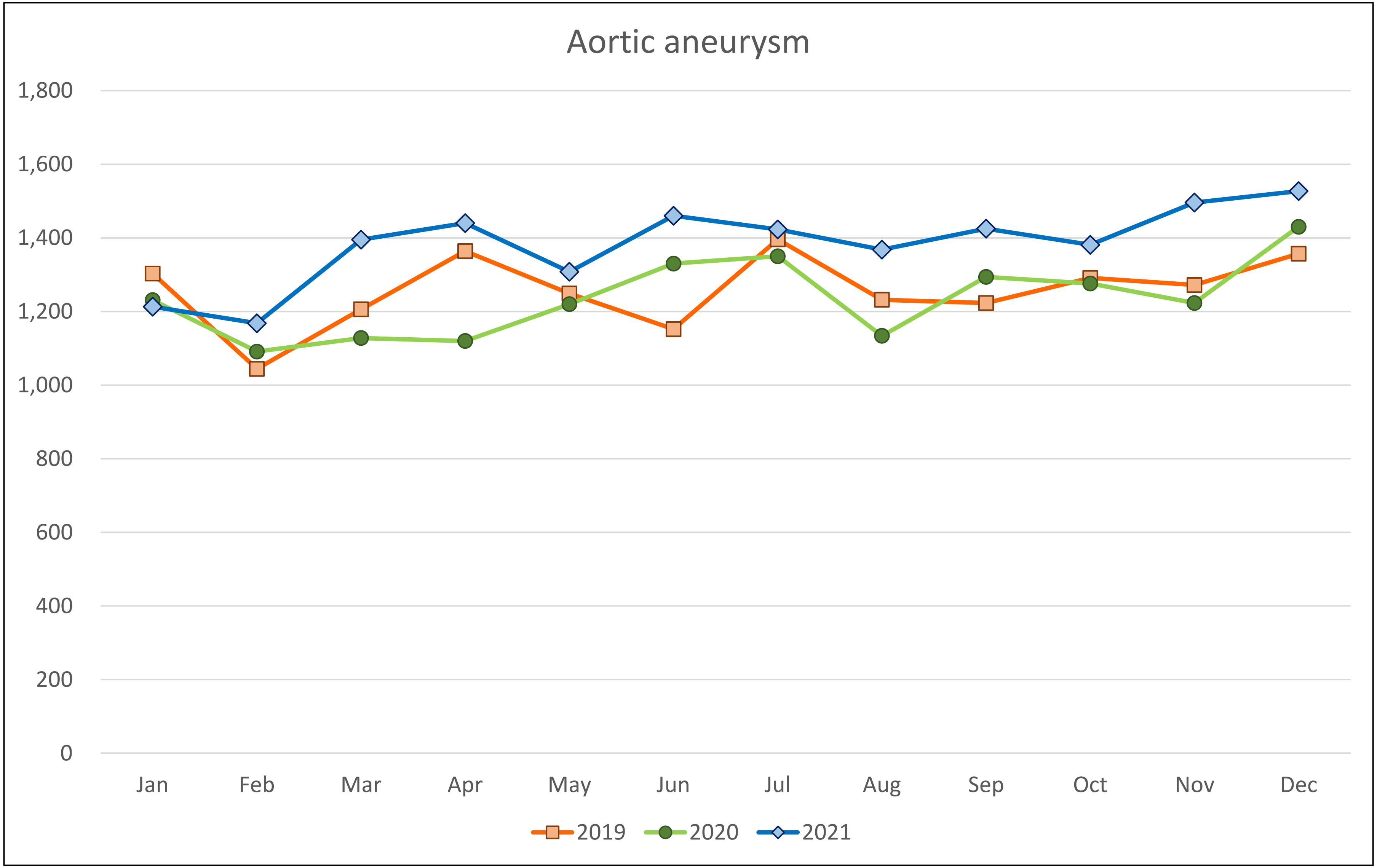
Aortic aneurysm
1,800
1,600
1,400
1,200
1,000
800
600
400
200
0
Jan
Feb
Mar
Apr
May
Jun
Jul
Aug
Sep
Oct
Nov
Dec
2019
2020
2021

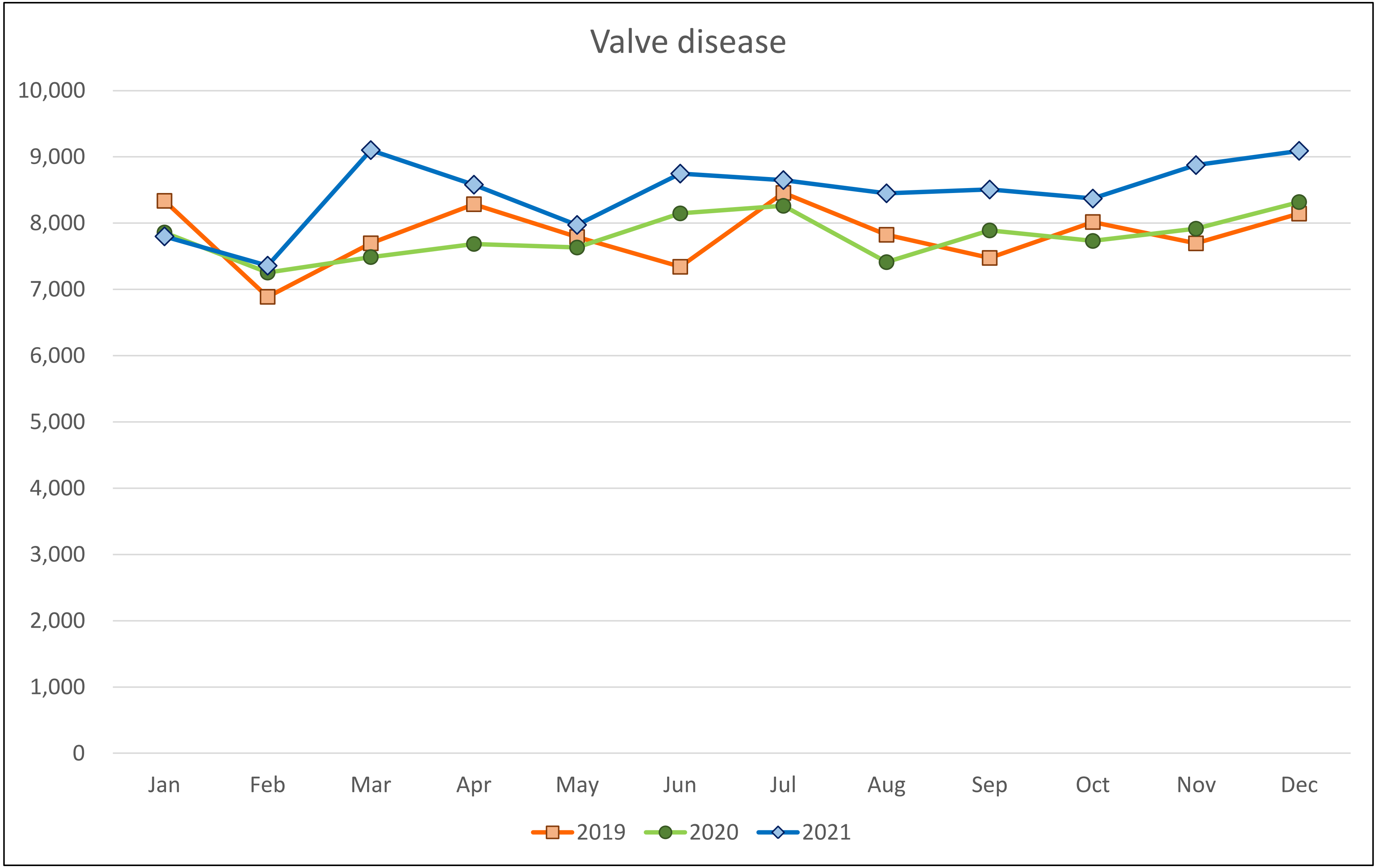
Valve disease
10,000
9,000
8,000
7,000
6,000
5,000
4,000
3,000
2,000
1,000
0
Jan
Feb
Mar
Apr
May
Jun
Jul
Aug
Sep
Oct
Nov
Dec
2019
2020
2021
